# Food, nutrition and diet in urban areas from low- and middle-income countries in the WHO European Region

**DOI:** 10.1017/S1368980022002051

**Published:** 2023-12

**Authors:** Gabriela Albuquerque, Nuno Lunet, João Breda, Patrícia Padrão

**Affiliations:** 1 EPIUnit - Instituto de Saúde Pública, Universidade do Porto, Porto 4099-002, Portugal; 2 Laboratório para a Investigação Integrativa e Translacional em Saúde Populacional (ITR), Porto, Portugal; 3 Departamento de Ciências da Saúde Pública e Forenses e Educação Médica, Faculdade de Medicina da Universidade do Porto, Porto, Portugal; 4 World Health Organization European Office for the Prevention and Control of Noncommunicable Diseases (NCD Office); 5 Faculdade de Ciências da Nutrição e Alimentação da Universidade do Porto, Porto, Portugal

## The WHO European Region (WHO-Europe): context and action plan

The WHO-Europe includes fifty-three Member States, embracing nearly 900 million people living in an area stretching from the Arctic Ocean in the north and the Mediterranean Sea in the south and from the Atlantic Ocean in the west to the Pacific Ocean in the east (Supplementary material 1). This vast territory reflects a diversity of populations, socio-economic, political and cultural circumstances and health challenges. The existence of large health inequalities (between and within subregions and the Member States) is one of the persistent issues, which has been aggravated by the coronavirus disease (COVID-19) and particularly affected low- and middle-income countries and vulnerable populations^(1)^. Moreover, a wide gap of representative diet surveys on nutritional status, dietary habits and food composition has been identified, particularly in Central and Eastern Europe and Central Asia^(2)^. This special issue of Public Health Nutrition includes a collection of articles from leading scientists and professionals developing research in the field of nutrition and public health in low- and middle-income countries, with special emphasis on countries in Eastern Europe and Central Asia.

Action in the WHO European Region is developed in close collaboration with all the Member States. The *WHO Strategy Action Plan for the Prevention and Control of Noncommunicable Diseases in the WHO European Region 2016–2025* ^(3)^ aims to avoid premature death and reduce the disease burden from non-communicable disease (NCD) by taking integrated action on risk factors (tobacco use, excessive alcohol consumption, unhealthy diet and physical inactivity) and their underlying determinants, improving the quality of life and making healthy life expectancy more equitable within and between the Member States (list of WHO Europe Member States available in supplementary material 1). It is aligned with global commitments to improve health and accelerate progress in Sustainable Development Goals (SDG) implementation, in particular SDG3 (ensure healthy lives and promote well-being for all at all ages)^(4)^. This commitment has been further strengthened by the adoption of the *WHO European Region SDG Roadmap*, a policy guidance that aimed to reinforce the need for high-level leadership for health and well-being and strong intersectoral mechanisms to address the many risk factors and determinants of health^(4)^. Thus, in recent years, several strategies have been carried out in the Region, aligned with these principles and with global WHO standards for healthy diets and requirements for the daily intake of nutrients such as fat, sugar, Na and K^(5–9)^, as well as for marketing to children of energy-dense, highly processed foods and beverages that are high in SFA and trans-fatty acids (TFA), free sugars and/or salt^(10)^.

## Diet and nutrition in WHO-Europe: burden and monitoring risk factors among adults

Worldwide, dietary risks were estimated to account for approximately 8 million deaths (23 %) and 188 million disability-adjusted life years (DALY) (16 %) among adults (2019), representing a marked increase since the 1990s^(11)^. Unhealthy diet is one of the main risk factors for premature deaths due to NCD in the WHO European Region^(1)^ and accounts for more than 10 % of DALY in countries in Central and Eastern Europe and Central Asia^(12)^. This high number of deaths and DALY is attributable to, among dietary risk factors, high Na intake, low consumption of whole grains, legumes and fruits, and high consumption of red meat. In addition, low consumption of nuts and seeds, vegetables, milk, seafood *n*-3 fatty acids, fibre, PUFA and calcium, and high consumption of processed meats, sugar-sweetened beverages and high intake of TFA are important dietary risk factors for death due to NCD^(11)^.

The current burden of morbidity and mortality has been induced by a series of demographic, social and economic transformations, which led to a global shift in patterns of health and disease^(13–15)^. Specifically, in Eastern Europe and Central Asia, the socio-economic development triggered by political shifts in the early 1990s, together with technology innovation, had impacts on food availability and consumption^(16,17)^. These changes encompassed a gradual replacement of traditional by more globalised eating patterns, through a decline in the consumption of fruits, vegetables, legumes and whole grains and a concomitant increase of animal-based and processed foods, more likely to be energy-dense, rich in fat, sugar and Na^(14,18,19)^. Increased availability of cheaper edible oils and fats, of lower-quality fatty acids profile, rich in TFA and SFA has also been recorded. Such changes have facilitated increased energy intake, which, coupled with the decreased activity patterns, seems to have resulted in the energy imbalance leading to the emergence of overweight, obesity and diet-related NCD^(14,19–25)^.

The WHO recommends that adults consume no more than 2 g of Na (approximately 5 g of salt) per d, to reduce the risk of CVD and NCD^(5)^. Reducing population intake of Na has a significant potential for health gains, being one of the most cost-effective measures in reducing the burden of NCD and improving population health^(26)^. As such, as part of the WHO-Europe strategy to reduce the burden of NCD, a global target of a 30 % reduction in population salt intake by 2025 was agreed upon, through the implementation and scaling up of comprehensive salt reduction strategies^(12)^. Although exemplary effort to tackle excess Na intake has been achieved across the WHO-Europe, average intakes were still above the recommendation, in 2017. At the time, over half the countries in the Region had national policies on salt reduction implemented (68 %)^(27)^, a potential marker of progress and commitment for scaling up monitoring. Nevertheless, progress falls far short of the recommendations and more concerted efforts are needed not only to develop effective salt reduction policies, programmes and interventions but also to conduct high-quality surveillance. One of the main challenges is the need for a unified source of the most up-to-date data on the population salt intake of every country in the Region.

Following such concerns, the aim of one systematic review in this special issue (PHN-RES-2021–1176) was to analyse the average population daily salt intake in the fifty-three Member States of the WHO European Region, based on the most up-to-date information available in published literature. Most countries in WHO/Europe (*n* 51, 96·0 %) reported salt intake above WHO recommended maximum levels. In 98 % of these countries, salt intake was higher among men, ranging between 4·29 and 18·51 g for men and 3·11 and 16·14 g for women. A comparison between subregions showed the lowest average salt intake in the Western and Northern European countries (lowest national average intake: 3·70 g, in Luxembourg), in contrast to the highest average salt intake, observed in Eastern European and Central Asian countries (highest national average intake: 17·24 g, in Kazakhstan and Kyrgyzstan). Although some heterogeneity was observed in measuring salt intake, the gold standard method (24-h urinary collection) was used in approximately 40 % of all the Member States. Despite the methodological limitations, the finding that overall salt intakes in WHO-Europe are currently above WHO recommendations is central to supporting the strengthening of the body of evidence on which policies to reduce salt intake can be developed and subsequently monitored.

Evaluations of national salt reduction programmes have shown that it is possible to reduce the salt intake of a population through a combination of measures, including comprehensive population awareness campaigns on diet change^(28)^. For these measures to be effective in low- and middle-income countries from the WHO European Region, it becomes of utmost importance to study the current level of awareness regarding the impact of high-salt diets on health within these populations. Another publication in this special issue (PHN-RES-2021-0613.R1) aimed to assess educational inequalities in high blood pressure awareness, treatment and control, patient advice on salt reduction, and salt knowledge, perceptions and consumption behaviours in nine Eastern European and Central Asian countries (Armenia, Azerbaijan, Belarus, Georgia, Kyrgyzstan, Republic of Moldova, Tajikistan, Turkey and Uzbekistan). Data were collected in cross-sectional, population-based nationally representative surveys of adults aged 25–65 years, using a multi-stage clustered sampling design (the WHO STEPwise approach to surveillance – STEPS survey^(29)^). High blood pressure awareness, treatment and control varied substantially by education. The coverage of patient advice on salt was less frequent among people with lower education, and those with untreated high blood pressure or unaware of their high blood pressure. The education gradient was evident in salt knowledge and perceptions of salt intake but not in salt consumption behaviours. Improved salt knowledge and perceptions were more prevalent among persons who received medical advice on salt reduction. In light of these findings, enhancements in public and patient knowledge and awareness of high blood pressure and associated risk factors are urgently needed, especially in targeting socio-economically disadvantaged groups, to alleviate the growing high blood pressure burden in low- and middle-income countries.

WHO Europe calls for a reduction, or ‘virtual elimination’ of industrially produced TFA (i-TFA), based on evidence that TFA consumption significantly increases the risk of CHD^(30)^. The *2003 WHO/FAO Technical Report Series 916* stated that intake of TFA should account for < 1 % of total energy intake^(31)^. More recent recommendations include setting legislative limits for the i-TFA content in foods (which is regarded as one of the most simple, cost-effective and universal public health interventions for reducing cardiovascular risk and improving diet quality^(32)^) and the replacement of TFA with unsaturated fats^(30,33)^. As a consequence, some countries defined regulations to limit the TFA content of food to 2 g per 100 g of fat, in practice eliminating the use of industrially produced TFA in cooking and food manufacture^(32)^. The Eurasian Economic Union^(34)^ and the European Union^(35)^ introduced similar legislation, to be in force, respectively in 2018 and 2021. As of 2017, 62 % of countries in the WHO European Region had implemented national policies limiting SFA and virtually eliminating industrially produced TFA in the food supply^(27)^.

Substantial health benefits have been recently observed globally in countries that have virtually eliminated TFA from their food supply^(36–39)^. Building on these findings, a study included in this special issue (PHN-RES-2021-0445.R1) explored the potential impact on population health if policies designed to reduce population TFA intake to < 1 % of the total energy value are successfully implemented in some countries in the WHO-Europe. Specifically, the authors estimated the projections of reductions in CVD-related deaths if TFA policies are adhered to in Armenia, Belarus, Kazakhstan, Kyrgyzstan and the Russian Federation, under three scenarios corresponding, respectively, to the end of the first, second and third year after the policy implementation. Data used for the projection exercise were based on estimates from natural experimental evidence from Denmark^(38)^, and data on national cardiovascular mortality rates used were collected from WHO and the Organization for Economic Co-operation and Development (OECD) datasets. Consistently in all scenarios, at least 5 deaths/100 000 were averted in each of the countries in the first year, with observed increases in the two subsequent years. The highest projected impacts in the high exposure scenario (third year) were seen in Kyrgyzstan (39 deaths/100 000) and the lowest in Armenia (24 deaths/100 000). From these findings, it becomes clear that, as recommended by the WHO, effective policies to reduce i-TFA have significant health gains. Better monitoring and surveillance systems are thus needed to evaluate the compliance with TFA reduction policies and the real effectiveness of such policies. This will provide insight on the progress and also guidance for strengthening potential additional actions towards the ultimate goal of the ‘virtual elimination’ of TFA at both national and regional levels.

## Diet and Nutrition in the WHO-Europe: monitoring and surveillance of risk factors among children

The needs to a) bridge the gap of diet and nutrition surveys in countries in Central Asia and Eastern Europe^(2)^, b) characterise NCD-related indicators and c) support the implementation of national surveys to strengthen the monitoring and surveillance of NCDs led to the implementation of several valuable initiatives across the WHO European Region. Examples include surveys on multiple risk factors – such as the household-based STEPS survey^(29)^ and single risk factors – such as the Childhood Obesity Surveillance Initiative (COSI). The COSI surveillance system aims to collect information on nutritional status, by routine and standardised measurement of body weight, height and related lifestyle behaviour (e.g. dietary habits, physical activity, screen time, sleep time and nutrition environments in schools) in nationally representative samples of primary school-aged children living in the WHO European Region^(40)^. This initiative has been contributing, in recent years, to the improved understanding of risk factors for childhood overweight and obesity in the Region, but further and continuous efforts are needed.

Based on data collected in the fourth round of the COSI Tajikistan (2016/17), one study included in the present special issue (PHN-RES-2021–0652) described the nutritional status and eating behaviours of a representative sample of Tajik children (*n* 3318) and explored the association between weight status and diet, family socio-economic status and residential urbanicity. The prevalence of thinness, overweight and obesity was 4·3 %, 7·6 % and 1·5 %, respectively. Regarding dietary intake, most children did not eat fresh fruit (66·4 %) or vegetables (56·9 %) daily, and 44·3 % of children reported a consumption of sugary soft drinks at least 4 d per week. Having a family with low or medium perceived wealth was associated with inadequate consumption of fresh fruit, while low and medium parental education level was associated with inadequate consumption of vegetables. These findings illustrate a low prevalence of childhood obesity in Tajikistan (2016/17), concurrently with one of the highest thinness prevalences observed in the WHO European Region and associated dietary behaviours. This reinforces the importance of the surveillance systems to closely track the effects of the nutritional transition occurring in the region, especially the double burden of nutrition, aggravated by the COVID-19 pandemic^(1)^.

Children’s exposure to marketing of foods and drinks high in SFA, TFA, free sugars and/or salt has been demonstrated to have an impact on children’s eating behaviour and body weight and is one of the core strategic focus of the WHO-Europe^(41)^. The study by Kontsevaya et al (PHN-RES-2021-0650) aimed to compare the frequency and healthfulness of food advertising to children and adolescents in four countries in WHO European Region (Russia, Turkey, Kazakhstan and Kyrgyzstan). The study was based on an adapted version of the WHO protocol ‘Monitoring food and beverage marketing to children via television and the Internet’^(42)^. It encompassed the analysis of 70 d of TV broadcasting (all channels available in each country). The mean number of advertisements per d varied from 89·9 in Kyrgyzstan to 584·0 in Russia. In all countries, most food and beverages did not comply with the recommended foods to be advertised to children^(41)^. For Turkey, nutritional information about all the advertised foods was available, and all product categories were able to be classified. For the remaining countries, the absence of nutritional information led to proportions of advertisements that could not be classified according to the WHO Nutrient Profile Model for Europe varying from 7 % to 20 %. Nevertheless, the main findings highlight that across these four countries, television food and beverage advertisements were predominantly for products exceeding WHO maximum thresholds for SFA, Na, and/or sugar for foods and beverages that are considered appropriate to be marketed to children.

The challenges addressed in this publication should be faced as potential opportunities to shift towards more equitable, resilient and sustainable food systems and food environments that enhance access to healthy food and population health outcomes across the Region^(43)^. We believe that this collection of articles largely contributes to increasing the availability of data on dietary risk factors for NCD in low- and middle-income countries in the WHO European Region, giving an overview of the progress in tackling NCD. We hope it is useful to assist countries in defining public health priorities according to their real needs.

## Supplementary material

For supplementary material accompanying this paper visit https://doi.org/10.1017/S1368980022002051. click here to view supplementary material
