# Supplementary material for: Food, nutrition and diet in urban areas from low- and middle-income countries in the WHO European Region
Source: Public Health Nutr. 2022 Sep 20;26(Suppl 1):s1–5. doi: 10.1017/S1368980022002051 (PMC10801371; doi:10.1017/S1368980022002051)
Supplement: Supplementary file 1 [file S1368980022002051sup001.docx]

**Food, nutrition and diet in urban areas from low and middle-income countries in the World Health Organization European Region**

**Supplementary material 1. List of World Health Organization (WHO)-Europe Member States**

Fifty-three countries integrate the WHO European Region:

| Albania | Greece | Portugal |
| --- | --- | --- |
| Andorra | Hungary | Republic of Moldova |
| Armenia | Iceland | Romania |
| Austria | Ireland | Russian Federation |
| Azerbaijan | Israel | San Marino |
| Belarus | Italy | Serbia |
| Belgium | Kazakhstan | Slovakia |
| Bosnia and Herzegovina | Kyrgyzstan | Slovenia |
| Bulgaria | Latvia | Spain |
| Croatia | Lithuania | Sweden |
| Cyprus | Luxembourg | Switzerland |
| Czechia | Malta | Tajikistan |
| Denmark | Monaco | Turkey |
| Estonia | Montenegro | Turkmenistan |
| Finland | Netherlands | Ukraine |
| France | North Macedonia | United Kingdom of Great Britain and Northern Ireland |
| Georgia | Norway |  |
| Germany | Poland | Uzbekistan |
